# Supplementary material for: An updated suite of viral vectors for in vivo calcium imaging using intracerebral and retro-orbital injections in male mice
Source: Nat Commun. 2023 Feb 4;14:608. doi: 10.1038/s41467-023-36324-3 (PMC9899252; doi:10.1038/s41467-023-36324-3)
Supplement: Supplementary file 1 — Supplementary Information [file 41467_2023_36324_MOESM1_ESM.pdf]

Supplementary Information   Grødem & Nymoen et al. 2023

The supplementary information contains supplementary figures S1-7 and table S1.

In addition, 7 supplementary videos are attached to the manuscript.

Figure S1:

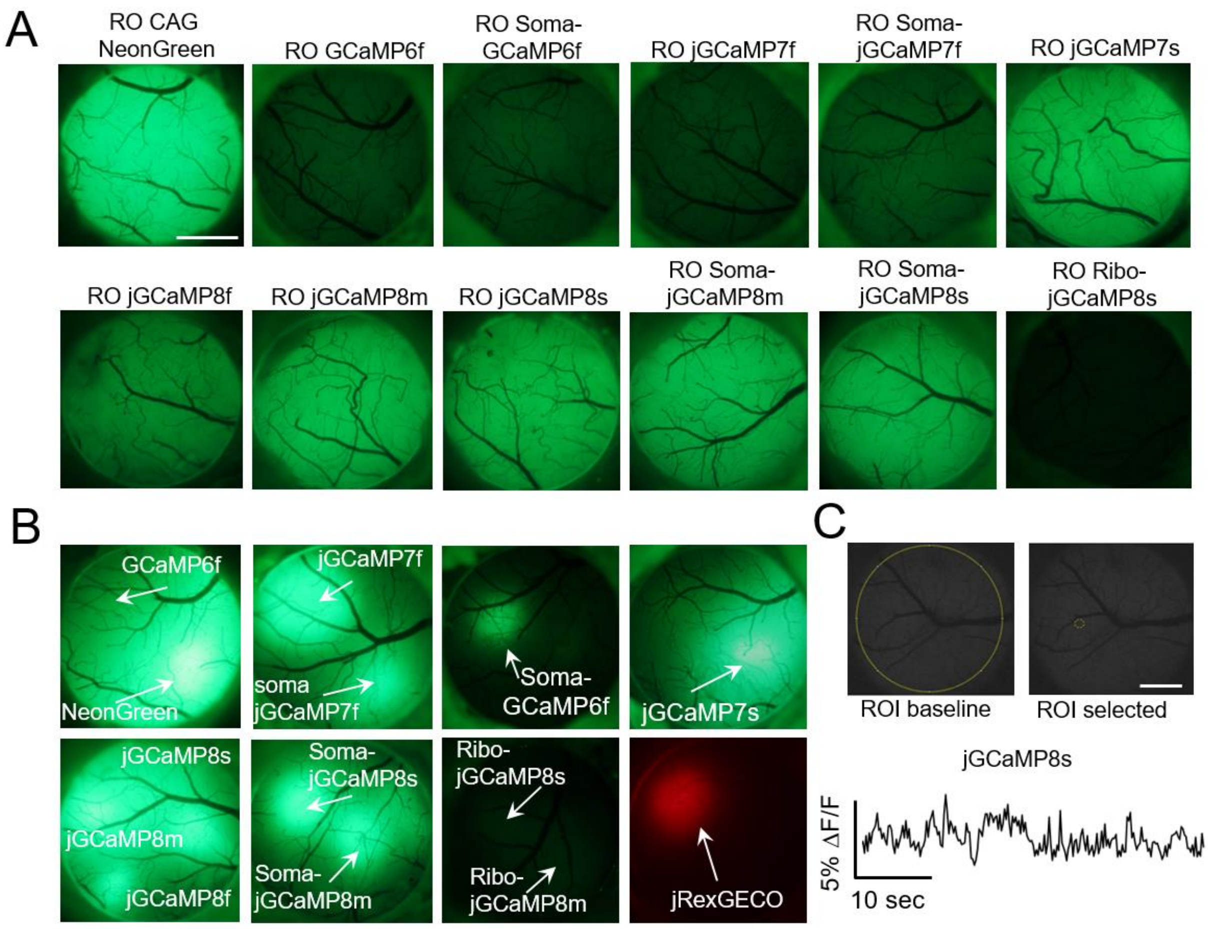

Figure S1: Overview of GECI screening using wide-field fluorescence microscopy. **A** shows RO injected animals, while **B** shows local virus injections. Scale bar (applies to images in A and B) indicates 1 mm. **C**: Wide-field imaging of spontaneous cortical activity did not indicate seizures from brain wide GECI expression. Example of a region of interest and Ca2+ trace. Scale bar indicates 1 mm.

Figure S2:

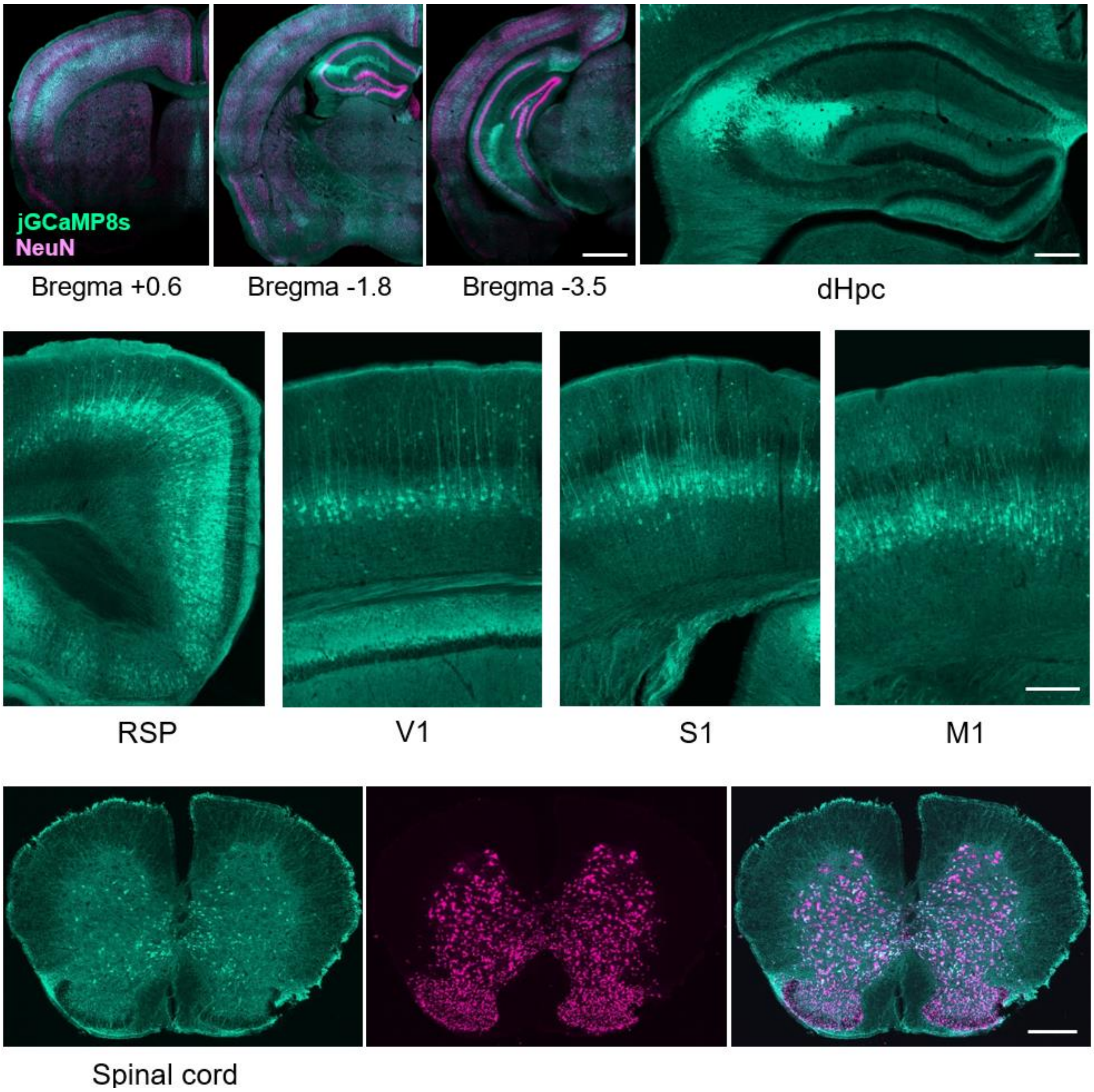

Figure S2: Examples of post-mortem histological samples from different brain areas and the spinal cord, stained with GFP and the neuronal marker NeuN. Scare bar for whole hemisphere images indicates 1 mm, for hippocampus and cortical areas 200 µm, and for spinal cord 250 µm. For A-C, the results were reproduced in n=3 mice.

Figure S3:

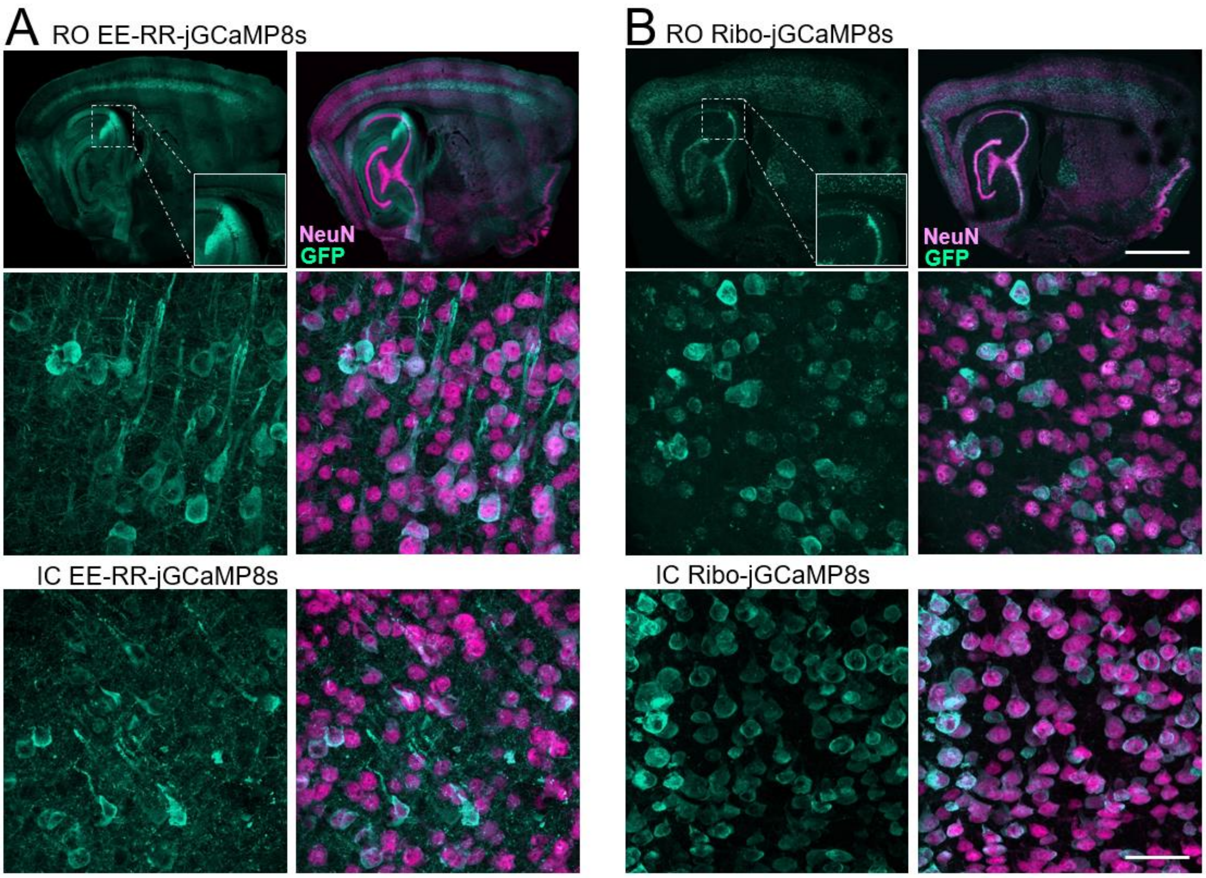

Figure S3: Histological verification of expression from soma-targeted expression of jGCaMP8. **A**: EE-RR soma targeting using RO or local virus injections. Upper panel shows a sagittal section of a mouse brain with hippocampal region CA2 highlighted. Lower panels show high-resolution images from primary visual cortex from both systemic and local virus injections. **B**: Same as for A, but using ribosome-tethered jGCaMP8s. Scale bars indicate 1 mm (upper panels) and 25 µm (lower panels). For A and B, The results were reproduced in two mice.

Figure S4:

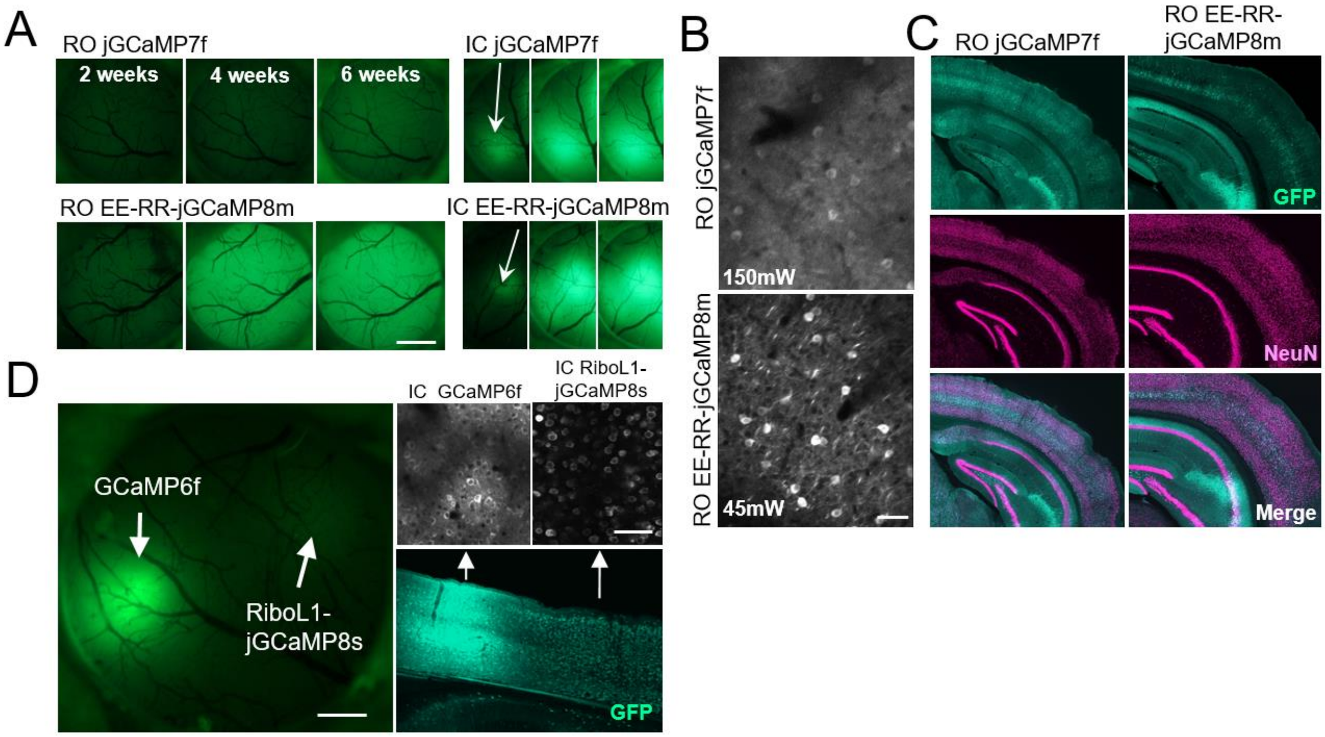

Figure S4: Examples of GECIs with different brightness *in vivo*. **A:** Wide field imaging examples of jGCaMP7f and EE-RR-jGCaMP8m at 2, 4 and 6 weeks after IC or RO injections. Scale bar indicates 1 mm. **B:** Example images from *in vivo* two-photon microscopy of jGCaMP7f and EE-RR-jGCaMP8m expressed by RO injection. jGCaMP7f is only detectable at very high laser power but shows comparable signal intensity to EE-RR-jGCaMP8s in post-mortem histological samples (**C**)**.** Scale bar indicates 30 µm**. D:** Within-subject difference in signal intensity following IC injections of GCaMP6f and RiboL1-jGCaMP8s in V1. Scale bars indicate 500 µm (wide-field image) and 50 µm. For A-C, the results were reproduced in n≥3 mice.

Figure S5:

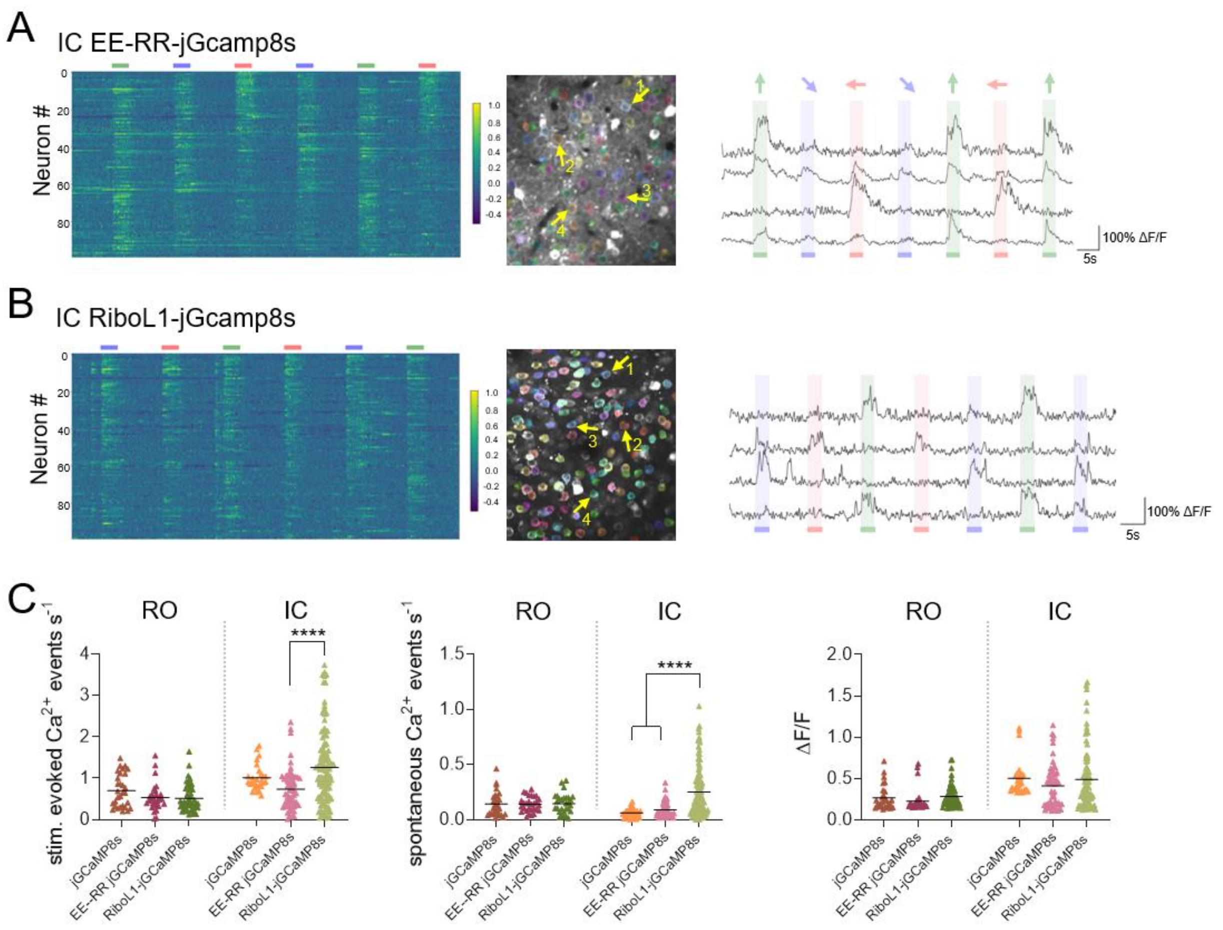

Figure S5: Cell responses and orientation tuning to visual stimuli. **A:** Responses of 100 neurons in V1 to drifting sinusoidal gratings of three different orientations 4 weeks after IC injection of EE-RR-jGCaMP8s (left panel). ROIs detected by suite2p (middle panel). Examples of tuning curves for single neurons (example ROIs indicated by yellow arrows) are shown in the right panel. **B:** Same as for A, using RiboL1-jGCaMP8s. **C:** Number of stimuli evoked (left panel) and spontaneous (middle panel) Ca^{2+} events per second for the different labelling strategies. Stimulus evoked ΔF/F cell responses averaged across stimulus trials for the different labelling strategies (right panel). Kruskal-Wallis test with Dunn's multiple comparisons (Tukey corrected), * indicates $p<0.05$. Source data are available as a Source Data file.

Figure S6:

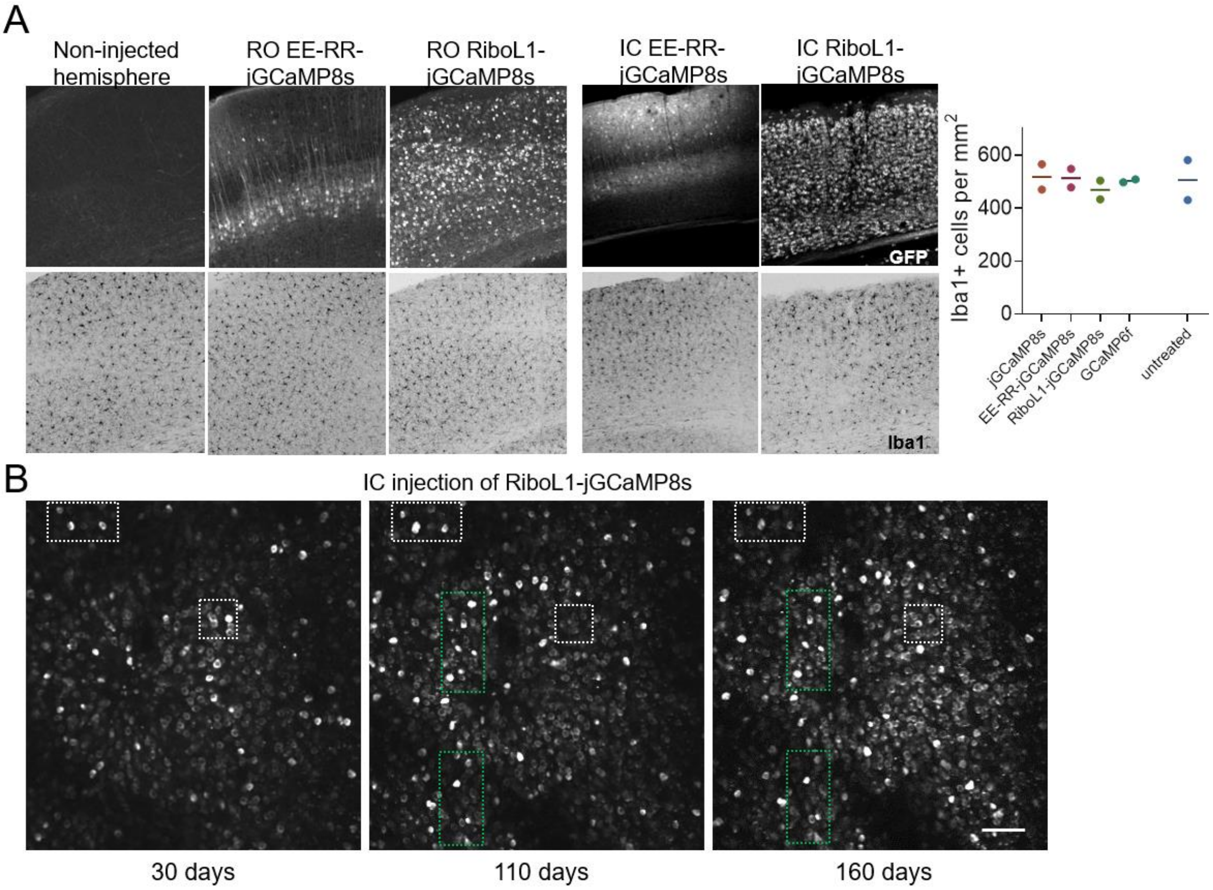

Figure S6: Verification of cell health and expression stability. **A:** Quantification of Iba1 positive cells in post-mortem histological samples from animals with EE-RR-jGCaMP8s and RiboL1-jGCaMP8s 10 weeks after RO or IC injections. **B:** *In vivo* verification of expression stability following IC injection of RiboL1-jGCaMP8s. Boxes indicate cells used to locate the same field of view across imaging days. Scare bar indicates 50 µm.

Figure S7:

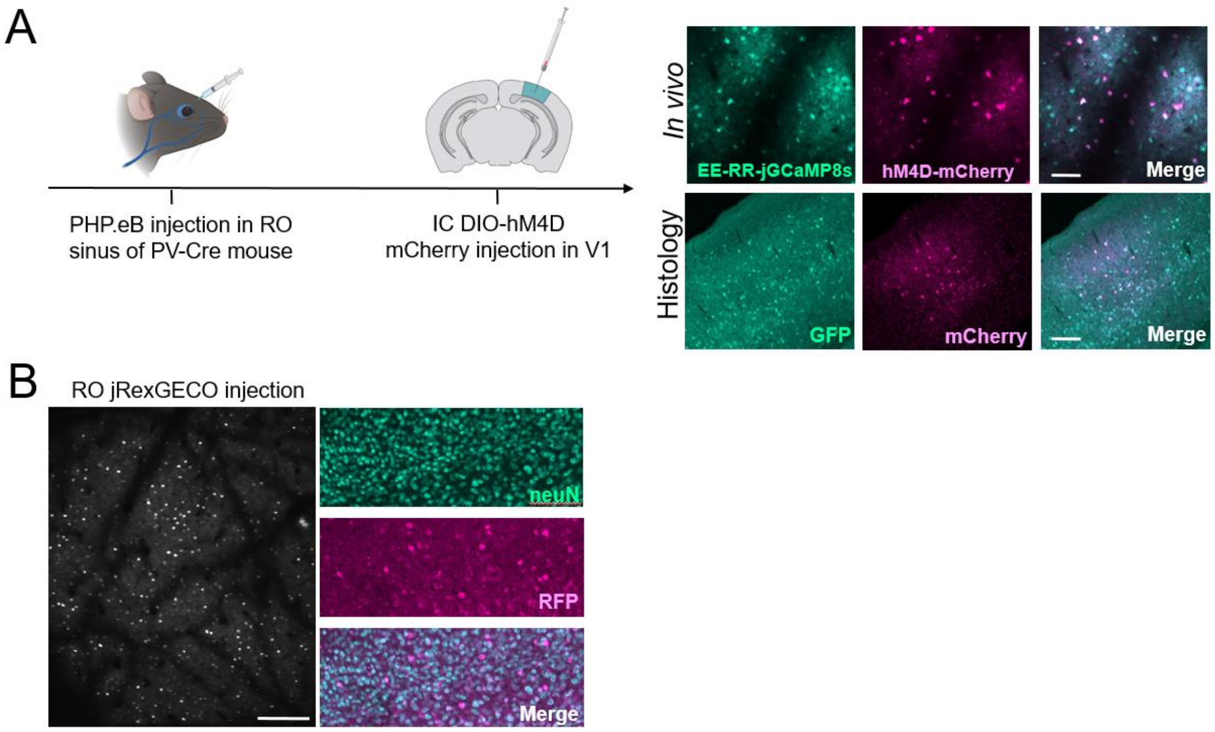

Figure S7: Applications of systemic GECI expression. **A:** experimental overview for GECIs expressed by RO injection and Cre-dependent hM4D expressed by local injection. Scale bars indicate 50 µm (in vivo) and 100µm (histology). The results were reproduced in two mice. Illustration created using BioRender. **B:** RO injection of the red-shifted GECI jREX-GECO1. Scale bar indicates 250 µm. The results were reproduced in n≥3 mice.

Table S1: Primers and synthesized DNA fragment sequences, 5’ to 3’:

Synthesized construct with flexible GC-ribo-linker (RiboL1)	cttggagagaagttaacagatgaagaggttgatgaaatgatcagggaagcagacatcgatggggatggtc<br>aggtaaactacgaagagtttgtacaaatgatgacagcgaagggggcagcggtgggtctggagggactg<br>ggggtagcggtggtagcggtgggacagggggaagcggcggtagtggtggaacaagcagcaaagtctca<br>cgcgacaccctgtacgaggcggtgcgggaagtcctgcacgggaaccagcgcaagcgccgcaagtttctg<br>gagacggtggagctgcagatcagcctgaagaactacgaccctcagaaggacaaacgtttctcgggcacc<br>gtcaggctcaagtccaccccacgccccaagttctcggtgtgcgttctgggggaccagcagcactgtgatgaa<br>gccaaggccgtggatatcccccacatggacatcgaggcgctcaagaaacttaacaaaaacaagaagttg<br>gtcaagaagctggctaagaagtacgatgcctttttggcctctgagtctctgattaagcagatcccacgtatcctg<br>ggcccaggcctaaacaaggctggcaagttcccctccctgctgacacacaatgaaaacatggtggccaaag<br>tggatgaggtgaaatcgacaatcaagttccagatgaagaaggtgctgtgtttggccgtcgctgttggccacgt<br>gaagatgaccgatgatgagctagtctacaacattcatc
Synthesized construct with rigid short ribo-linker (RiboL2)	cttggagagaagttaacagatgaagaggttgatgaaatgatcagggaagcagacatcgatggggatggtc<br>aggtaaactacgaagagtttgtacaaatgatgacagcgaagctggaggccgaagcagcagcaaaggag<br>gctgccgcgaaggaagcagcggccaaggaagcagcagcgaaggccctagagagcagcaaagtctca<br>cgcgacaccctgtacgaggcggtgcgggaagtcctgcacgggaaccagcgcaagcgccgcaagtttctg<br>gagacggtggagctgcagatcagcctgaagaactacgaccctcagaaggacaaacgtttctcgggcacc<br>gtcaggctcaagtccaccccacgccccaagttctcggtgtgcgttctgggggaccagcagcactgtgatgaa<br>gccaaggccgtggatatcccccacatggacatcgaggcgctcaagaaacttaacaaaaacaagaagttg<br>gtcaagaagctggctaagaagtacgatgcctttttggcctctgagtctctgattaagcagatcccacgtatcctg<br>ggcccaggcctaaacaaggctggcaagttcccctccctgctgacacacaatgaaaacatggtggccaaag<br>tggatgaggtgaaatcgacaatcaagttccagatgaagaaggtgctgtgtttggccgtcgctgttggccacgt<br>gaagatgaccgatgatgagctagtctacaacattcatc
Synthesized construct with rigid long ribo-linker (RiboL3)	cttggagagaagttaacagatgaagaggttgatgaaatgatcagggaagcagacatcgatggggatggtc<br>aggtaaactacgaagagtttgtacaaatgatgacagcgaaggctgaggctgctgctaaagaagccgccgc<br>taaagaggcagcagccaaagaagctgctgccaaggctctggaagctgaagccgcagcaaaagaagca<br>gccgcaaaggaggctgccgcaaaagaggccgctgcaaaggcaagcagcaaagtctcacgcgacaccc<br>tgtacgaggcggtgcgggaagtcctgcacgggaaccagcgcaagcgccgcaagtttctggagacggtgg<br>agctgcagatcagcctgaagaactacgaccctcagaaggacaaacgtttctcgggcaccgtcaggctcaa<br>gtccaccccacgccccaagttctcggtgtgcgttctgggggaccagcagcactgtgatgaagccaaggccg<br>tggatatcccccacatggacatcgaggcgctcaagaaacttaacaaaaacaagaagttggtcaagaagct<br>ggctaagaagtacgatgcctttttggcctctgagtctctgattaagcagatcccacgtatcctgggcccaggcc<br>taaacaaggctggcaagttcccctccctgctgacacacaatgaaaacatggtggccaaagtggatgaggtg<br>aaatcgacaatcaagttccagatgaagaaggtgctgtgtttggccgtcgctgttggccacgtgaagatgacc<br>gatgatgagctagtctacaacattcatc
Axon-jRex-Geco synthesized fragment	tgctagcaaggatccacccgccaccatgctgtgctgtatgagaagaaccaaacaggttgaaaagaatgat<br>gaggaccaaaagattatggtcgactcttcacgtcgtaagtggaataaggcaggtcacgcagtcagagctat<br>aggtcggctgagctcacgttgggtt
Axon-GCaMP synthesized fragment	cacagtcgtagctagaggatccacccgccaccatgctgtgctgtatgagaagaaccaaacaggttgaaaa<br>gaatgatgaggaccaaaagattATGCATCATCACCATCATCACACGCGTCGCAAGA<br>AGACCTTCAAGGAGGTGGCCACCG
FLEX-RiboL1-jGCaMP (5’ NheI) fwd (cloning primer)	TAAGCAGCTAGCCCGCCACCATGCATCATC
FLEX-RiboL1-jGCaMP rev (cloning primer)	TACTATGGCGCGCCCTAATACAGA
Forward sequencing primer for jGCaMP plasmids	tcgtgtcgtgcctgagagcg
Reverse sequencing primer for jGCaMP plasmids	cagcgtatccacatagcgtaaa
Forward sequencing primer for AAV-hSyn-JRex-GECO	tcgtgtcgtgcctgagagcg
Reverse sequencing primer for AAV-hSyn-JRex-GECO	cagcgtatccacatagcgtaaa
Forward ITR qPCR primer	ggaacccctagtgatggagtt
Reverse ITR qPCR primer	cggcctcagtgagcga
