# Supplementary material for: An updated suite of viral vectors for in vivo calcium imaging using intracerebral and retro-orbital injections in male mice
Source: Nat Commun. 2023 Feb 4;14:608. doi: 10.1038/s41467-023-36324-3 (PMC9899252; doi:10.1038/s41467-023-36324-3)
Supplement: Supplementary file 3 — Description of Additional Supplementary Files [file 41467_2023_36324_MOESM3_ESM.pdf]

Description of Additional Supplementary Files

File Name: Supplementary Movie 1
Description: Examples of neurons in V1 expressing jGCaMP8m after RO injection. Video averaged across 20 frames.

File Name: Supplementary Movie 2
Description: Examples of neurons in V1 expressing EE-RR-jGCaMP8m after RO injection. Video averaged across 20 frames.

File Name: Supplementary Movie 3
Description: Examples of neurons in V1 expressing EE-RR-jGCaMP8m after RO injection. Similar to Video S2, but with higher optical zoom. Video averaged across 20 frames.

File Name: Supplementary Movie 4
Description: Examples of neurons in V1 expressing EE-RR-jGCaMP8s after IC injection. Video averaged across 20 frames.

File Name: Supplementary Movie 5
Description: Examples of neurons in V1 expressing Ribo-jGCaMP8m after IC injection. Video averaged across 20 frames.

File Name: Supplementary Movie 6
Description: Examples of neurons in V1 expressing Ribo-jGCaMP8m after IC injection. Similar to Video S5, but with higher optical zoom. Video averaged across 20 frames.

File Name: Supplementary Movie 7
Description: Examples of neurons in V1 expressing RiboL1-jGCaMP8s after IC injection. Video averaged across 20 frames.
