# Supplementary material for: An updated suite of viral vectors for in vivo calcium imaging using intracerebral and retro-orbital injections in male mice
Source: Nat Commun. 2023 Feb 4;14:608. doi: 10.1038/s41467-023-36324-3 (PMC9899252; doi:10.1038/s41467-023-36324-3)
Supplement: Supplementary file 11 — Reporting Summary [file 41467_2023_36324_MOESM11_ESM.pdf]

nature portfolio

Corresponding author(s): Kristian Kinden Lensjø

Last updated by author(s): Jan 19, 2023

Reporting Summary

Nature Portfolio wishes to improve the reproducibility of the work that we publish. This form provides structure for consistency and transparency in reporting. For further information on Nature Portfolio policies, see our Editorial Policies and the Editorial Policy Checklist.

Statistics

For all statistical analyses, confirm that the following items are present in the figure legend, table legend, main text, or Methods section.

n/a	Confirmed	
☐	☒	The exact sample size (*n*) for each experimental group/condition, given as a discrete number and unit of measurement
☐	☒	A statement on whether measurements were taken from distinct samples or whether the same sample was measured repeatedly
☐	☒	The statistical test(s) used AND whether they are one- or two-sided<br>*Only common tests should be described solely by name; describe more complex techniques in the Methods section.*
☐	☒	A description of all covariates tested
☐	☒	A description of any assumptions or corrections, such as tests of normality and adjustment for multiple comparisons
☐	☒	A full description of the statistical parameters including central tendency (e.g. means) or other basic estimates (e.g. regression coefficient) AND variation (e.g. standard deviation) or associated estimates of uncertainty (e.g. confidence intervals)
☐	☒	For null hypothesis testing, the test statistic (e.g. *F*, *t*, *r*) with confidence intervals, effect sizes, degrees of freedom and *P* value noted<br>*Give P values as exact values whenever suitable.*
☒	☐	For Bayesian analysis, information on the choice of priors and Markov chain Monte Carlo settings
☒	☐	For hierarchical and complex designs, identification of the appropriate level for tests and full reporting of outcomes
☒	☐	Estimates of effect sizes (e.g. Cohen's *d*, Pearson's *r*), indicating how they were calculated

Our web collection on statistics for biologists contains articles on many of the points above.

Software and code

Policy information about availability of computer code

Data collection	MScan (MCS, Sutter Instrument) version 3.0.<br>Fusion (Andor) version 2.2
Data analysis	Fiji /ImageJ(NIH) versions 1.53 o, p and t<br>Python 3.0 - custom code available at https://github.com/frederikrogge/calcium-sensor-analysis<br>Suite2p - available at github.com/MouseLand/suite2p<br>CellPose - available at github.com/MouseLand/cellpose<br>PsychoPy version 3.2.3<br>Graphpad Prism version 8.0.2

For manuscripts utilizing custom algorithms or software that are central to the research but not yet described in published literature, software must be made available to editors and reviewers. We strongly encourage code deposition in a community repository (e.g. GitHub). See the Nature Portfolio guidelines for submitting code & software for further information.

Data

Policy information about availability of data

All manuscripts must include a data availability statement. This statement should provide the following information, where applicable:

- Accession codes, unique identifiers, or web links for publicly available datasets
- A description of any restrictions on data availability
- For clinical datasets or third party data, please ensure that the statement adheres to our policy

All plasmids used have been uploaded to Addgene, and antibodies used are listed with their corresponding RRID in Table 3. Source data used for analysis in Fig 5 and S5 are provided with this paper. Original raw data from imaging experiments will be available in a public repository with time, and are available upon reasonable request to the authors.

Human research participants

Policy information about studies involving human research participants and Sex and Gender in Research.

Reporting on sex and gender	NA
Population characteristics	NA
Recruitment	NA
Ethics oversight	NA

Note that full information on the approval of the study protocol must also be provided in the manuscript.

Field-specific reporting

Please select the one below that is the best fit for your research. If you are not sure, read the appropriate sections before making your selection.

☒ Life sciences ☐ Behavioural & social sciences ☐ Ecological, evolutionary & environmental sciences

For a reference copy of the document with all sections, see nature.com/documents/nr-reporting-summary-flat.pdf

Life sciences study design

All studies must disclose on these points even when the disclosure is negative.

Sample size	The data in our manuscript is mainly qualitative, where quality of virus injections were the main parameter that could interfere with our interpretation. We have replicated our results in 3-5 mice.
Data exclusions	No data were excluded. One mouse showed partial blindness a few weeks after virus injection but the expression did not differ from its duplicate and the mouse was therefore included.
Replication	All viruses were tested in two mice with systemic virus injections, and in two mice with local virus injections. For the GECIs that were successful to use with both injection techniques, we have replicated the results in 3-5 animals.<br>The plasmids were sequenced and the viruses were tested in cell culture prior to injections.
Randomization	Virus injections were performed in litter mates randomly assigned to a construct. The researcher performing surgeries and imaging was not the same as doing virus injections.
Blinding	Blinding was performed for post-mortem histology, and for analysis shown in Figure 5 A-C. Other than these experiments, all data acquisition was performed with identical settings unless described otherwise (e.g. laser power output, PMT sensitivity) and blinding was therefore not relevant.

Reporting for specific materials, systems and methods

We require information from authors about some types of materials, experimental systems and methods used in many studies. Here, indicate whether each material, system or method listed is relevant to your study. If you are not sure if a list item applies to your research, read the appropriate section before selecting a response.

Materials & experimental systems

n/a	Involved in the study	
☐	☒	Antibodies
☒	☐	Eukaryotic cell lines
☒	☐	Palaeontology and archaeology
☐	☒	Animals and other organisms
☒	☐	Clinical data
☒	☐	Dual use research of concern

Methods

n/a	Involved in the study	
☒	☐	ChIP-seq
☒	☐	Flow cytometry
☒	☐	MRI-based neuroimaging

Antibodies

Antibodies used

Antibodies with RRID and dilutions used are listed in detail in Table 3.

Validation

A10262-Antibody specificity was demonstrated by detection of different targets fused to GFP tag in transiently transfected lysates tested. Relative detection of GFP tag was observed across different proteins fused with GFP in H3-GFP (Lane 3-5) and p65-GFP (Lane 6). GFP-variant, YFP is also being detected in His-p65-YFP lysate (Lane 7), using Anti-GFP Polyclonal Antibody (Product # A10262) in Western Blot.
Ab177487-verification described using a wide range of methods at https://www.abcam.com/neun-antibody-epr12763-neuronal-marker-ab177487.html
AB8181 - In 293HEK cells transfected with cds plasmid detects a band of 55 kDa by Western blot. It also detects tdTomato in brain sections by IHC. This antibody is specific for tdTomato and mCherry proteins. It does not cross-react to GFP (green fluorescent protein).
PV27 - Antiserum PV27 labels a subpopulation of neurons in the normal brain with high efficiency (Fig. 1a), but does not stain the brain of parvalbumin knock-out mice (Fig. 1b). The antiserum PV27 recognizes the antigen at 12 kDa after SDS-gel electrophoretic separation of brain extracts
PA5-18039 - vverification described using a wide range of methods at at https://www.thermofisher.com/antibody/product/IBA1-Antibody-Polyclonal/PA5-18039

Animals and other research organisms

Policy information about studies involving animals; ARRIVE guidelines recommended for reporting animal research, and Sex and Gender in Research

Laboratory animals

Male C57/BL6J mice were purchased from Janvier Labs, delivered at 4 weeks of age, and experiments began at 6 weeks of age.

Wild animals

No wild animals were used in the study.

Reporting on sex

Only male mice were used in this study. In separate experiments we have repeated the findings in female mice, as would be expected.

Field-collected samples

No field-collected samples were used in this study.

Ethics oversight

Mattilsynet (Animal research Authority of Norway), Project ID 14680

Note that full information on the approval of the study protocol must also be provided in the manuscript.
